# Understanding the Relationship Between Grit and Foreign Language Performance Among Middle School Students: The Roles of Foreign Language Enjoyment and Classroom Environment

**DOI:** 10.3389/fpsyg.2019.01508

**Published:** 2019-07-05

**Authors:** Hongjun Wei, Kaixuan Gao, Wenchao Wang

**Affiliations:** ^1^School of Foreign Languages, Shihezi University, Shihezi, China; ^2^Faculty of Psychology, Beijing Normal University, Beijing, China

**Keywords:** grit, foreign language enjoyment, classroom environment, foreign language performance, positive psychology, second language learning

## Abstract

**Objective:**

This study aims to examine the effect of grit on foreign language performance (FLP) among middle school students. A mediated moderation model was constructed to assess the mediating role of foreign language enjoyment (FLE) and the moderating role of classroom environment (CE) in the relationship between grit and FLP.

**Methods:**

The study adopted the Grit Scale-Short Version, the Chinese Version of the FLE Scale, and the English CE Inventory to investigate 832 middle school students, and recorded the students’ FLP in their final exam after 1 month. Correlation and regression analyses were used to evaluate the relationships between grit, FLE, CE, and FLP.

**Results:**

The results indicated that grit positively affected FLP. In addition, FLE mediated the relationship between grit and FLP, and CE moderated the relationship between grit and FLE, and between grit and FLP.

**Conclusion:**

Grit not only directly promotes the FLP of middle school students but also indirectly improves FLP by promoting FLE. In addition, the impact of grit on FLE and FLP increases in a positive CE.

## Introduction

With the development of Positive Psychology (PosPsy), researchers have gradually realized the important role of PosPsy in the study of second language acquisition (SLA). Seligman and Csikszentmihalyi (2014) identified the goals of PosPsy and outlined the three pillars in PosPsy research: positive individual traits, positive experiences, and positive institutions. In *Positive Psychology Perspectives on Foreign Language Learning and Teaching*, Gabryś-Barker (2016) emphasized that learners’ personality traits, positive emotions, and learning environments are three important aspects that influence foreign language learners’ academic performance.

Today, China has more than 176.7 million people learning foreign languages (usually English), which constitutes the largest foreign language learner group in the world (You and Dörnyei, 2016). Foreign language is a compulsory course for every Chinese middle school student, and foreign language performance (FLP) is an important index used to assess whether middle school students can enter a good university. Therefore, it is necessary to study the influencing factors of middle school students’ FLP from the PosPsy research perspective.

### Grit Affects FLP

Grit is a self-regulation and non-cognitive personality trait composed of two underlying factors: persistence and long-term consistency of interests (Duckworth et al., 2007). Duckworth et al. (2007) believe that grit is the ability to motivate individuals to work hard and stick to long-term goals and that it is a kind of persistence in overcoming challenges and achieving large goals.

Grit has been found to enhance academic performance in school-aged adolescents from diverse cultural contexts (e.g., see a meta-analysis by Credé et al., 2017). Grittier students achieve higher grade point averages, higher levels of educational attainment, and greater success in scholastic competitions (Duckworth et al., 2007; Duckworth and Quinn, 2009; Cross, 2014). Moreover, individuals with a high level of grit can control the time and way of learning and reported more learning engagement, making them more likely to achieve excellent academic performance (Wolters and Hussain, 2015; Aparicio et al., 2017). In the field of foreign language learning, some studies have found that grit can promote academic performance. Additionally, previous studies have found that consistent practice of language skills leads to procedural knowledge and automatization (DeKeyser, 2007). Lake (2015) found that grit has a strong relationship to the measure of persistent effort for learning a second language. MacIntyre (2016) believe that grit is one of the most important positive personality traits that affect second language learning.

While extensive studies have documented that grit can improve academic performance, the predictive mechanisms for grit in relation to academic performance remain unclear. According to Caspi et al. (2005), the relationship between personality traits like grit and academic achievement might emerge as consequences of both indirect and direct effects of personality on achievement. Foreign language enjoyment (FLE) and classroom environment (CE) may play mediating and moderating roles, respectively.

### The Mediating Role of FLE

The role of emotion in SLA has received extensive attention. As a personality trait, grit may affect many academic emotions, such as depression and anxiety (Datu et al., 2018). In addition, some studies have found that grit is positively associated with positive emotions, such as happiness, the pursuit of goals, and subjective well-being (Singh and Jha, 2008; Duckworth et al., 2009; Duckworth and Gross, 2014). Given the designation of grit as a personality trait (Duckworth et al., 2007), gritty individuals are more likely to have positive academic emotions, such as FLE. As a typical and common positive emotion experienced by foreign language learners, FLE has attracted increasing scholarly attention (Dewaele and MacIntyre, 2014; Pavelescu and Petric, 2018).

According to Bandura’s social cognitive theory of self-regulation, self-regulatory systems consist of three principal subfunctions: self-monitoring, self-judgment, and self-reactive. In the stage of self-reactive, individuals will evaluate their own behaviors and then generate emotional experiences, such as self-satisfaction, pride, and self-criticism (Bandura, 1991). Individuals with high grit are able to perform better in academic behaviors, so they are more likely to have good self-reactions and positive emotions, such as FLE. In addition, previous studies have found that individuals with high grit levels tend to make positive attributions, have a more optimistic growth mindset, and have more positive emotions (Duckworth et al., 2009; Hill et al., 2016), which means that high-grit individuals may have more FLE.

Foreign language enjoyment is a concept that resonates with the emerging field of PosPsy and, more specifically, the broaden-and-build theory (Fredrickson, 2001). The broaden-and-build theory emphasizes that positive emotion, such as enjoyment, can broaden individuals’ thought-action repertoires and build their psychological resiliency and personal resources (Fredrickson, 2001; Oxford, 2015). Positive emotions are also conducive to individual exploration, allowing individuals to acquire new experiences and learn effectively (Dewaele and MacIntyre, 2014). Furthermore, according to the control-value theory, enjoyment is a positive and emotion-centered activity, one that positively impacts learners’ academic performance(Pekrun et al., 2007). FLE boosts foreign language learning as it encourages learners to be creative and explore an unfamiliar language (Dewaele and MacIntyre, 2016). Many previous studies have also found that enjoyment is typically linked with less anxiety and higher scholastic attainment (Brantmeier, 2005; Dewaele and MacIntyre, 2014; Dewaele and Dewaele, 2017). Dewaele and Alfawzan (2018) investigated the effect of FLE and foreign language classroom anxiety (FLCA) on FLP and found that the positive effect of FLE on performance was stronger than the negative effect of FLCA. From the above, the present study hypothesized that FLE may play a mediating role in how grit impacts FLP.

### The Moderating Role of CE

Although many previous studies found that grit scores are relatively strongly related to success, as suggested by the initial findings by Duckworth and colleagues (e.g., Duckworth et al., 2007; Duckworth and Gross, 2014; Strayhorn, 2014), but many others (e.g., Macnamara et al., 2014; Paunesku et al., 2015; Steinmayr et al., 2018) have failed to find strong relationships between grit scores and indicators of success. One possible reason is that moderating variables affect how grit impacts academic performance, making the relationship different in different states.

According to Lewin (1936)’s field theory, an individual’s behavior (B) is a function of the person (P) and the person’s environment (E). Most of the foreign language learning of middle school students takes place in class, making CE an important factor affecting their foreign language learning (Fraser, 2007). CE, also called class climate or class atmosphere, generally refers to the sum of various physical, social, and psychological factors that influence the development of teaching activities, quality, and effect (Fan and Dong, 2005).

Many previous studies have found that a positive CE can promote students’ FLP (Baek and Choi, 2002; Patrick et al., 2007). However, few studies have examined how CE interacts with individual personality traits and emotions to predict students’ FLP. In fact, in the view of PosPsy, a positive environment is conducive to fully realizing the advantages of individual positive personality traits (Seligman and Csikszentmihalyi, 2014). Highly gritty students are more likely to be fully engaged in learning and to achieve better academic performance in a foreign language in a positive CE versus a negative one. In a negative CE, even if students are willing to persist in learning, the interference of the outside environment may make students fail to reach the expected goal, despite their efforts. Therefore, CE may play a moderating role in the influence of grit on FLP. Similarly, academic enjoyment is a positive emotion acquired by students in foreign language learning. In a negative CE, students struggle to experience FLE, even if they make great efforts to study, which means that even high levels of grit may not promote academic enjoyment.

### Present Study

In recent years, many linguists have recognized the importance of improving learners’ grit and positive emotions to improve their language learning. In addition, increasingly more teachers have recognized the vital role played by positive classrooms (MacIntyre and Mercer, 2014). However, there is still a lack of existing research that examines how these three factors interact with each other and influence the mechanism of foreign language learning. This study examined the impact of grit on FLP from the perspective of PosPsy, and investigated the mediating effect of FLE and the moderating effect of CE.

## Materials and Methods

### Participants and Procedures

Participants in this study were 832 middle school students aged 11–16 years (M = 13.27; SD = 1.01); 463 were female, and 369 were male. In addition, the majority of participants (91.4%) belonged to the Han ethnic group, which is the majority ethnic group in China.

Prior to data collection, ethical approval for the study was obtained by Shihezi University. The experimenter contacted three middle schools located in Xinjiang and Anhui Province, China. After obtaining permission from school principals, informed consent forms were given to emerging adults who attended the classes. Given that all participants in the current study were juveniles under age 18, written informed consent was obtained from the parents of all participants prior to the survey. During school hours, a trained experimenter provided standardized instructions, and students were asked to complete the questionnaires during a 20-min period in the classroom. All the participants were asked to complete the measures that assessed the grit scale, FLE scale, and CE scale. One month later, the students took the final exam, and the foreign language scores of the final exam were obtained as indicators to measure FLP.

### Measures

#### Grit Scale

Grit was measured using the eight-item Grit Scale-Short Version (Grit-S; Duckworth and Quinn, 2009), which was validated for Chinese populations by a previous study and showed adequate construct and criterion validity (Li J. et al., 2018). Sample items included: “Setbacks do not discourage me” or “I finish whatever I begin.” Participants were asked to rate each item from 1 (not like me at all) to 5 (very much like me) on a Likert-type scale. The average score of eight items (including 4 reverse-scored items) was calculated to yield the value of grit, with a higher value indicating greater grit. In the current study, Cronbach’s alpha was 0.83.

#### Chinese Version of the Foreign Language Enjoyment Scale

Foreign language enjoyment was measured using the Chinese Version of the Foreign Language Enjoyment Scale (Li C. et al., 2018). The original FLE Scale was developed by Dewaele and MacIntyre (2016). The revised Chinese Version of the FLE Scale included 11 items (e.g., “In class, I feel proud of my accomplishments”). Participants were asked to rate each item on a Likert scale ranging from 1 (Strongly disagree) to 5 (Strongly agree). In the present study, this scale had good internal consistency (α = 0.81).

#### English Classroom Environment Inventory

The English Classroom Environment Inventory developed by Liu and Liu (2010) was used to assess CE. The inventory is a 25-item instrument that assesses the English CE of middle school students (e.g., “Our English teacher likes students to ask questions at any time”). The items are rated on a 5-point Likert scale ranging from 1 (Almost never) to 5 (Almost always). In the present study, this scale had good internal consistency (α = 0.82).

#### Foreign Language Performance

One month later, the students who participated in the previous survey completed a final exam organized by the school uniformly. From this standardized exam, we obtained their foreign language scores. The exam materials were jointly established by a team of middle school English teachers. Students from three grades were involved in this study, and thus three different exam materials were adopted. The full score of each test was 150, and each test contained six sessions: listening, single choice, reading comprehension, cloze test, translation, and writing. The total score of translation and writing was 30, and the scores of these two parts were obtained from the average score of two raters. The rest of the scores were choice questions with only one correct answer. In order to evaluate the applicability of the test materials, we analyzed each test, and examined four indicators including difficulty value, discrimination, reliability, and validity. The results showed that the indicators of each test were acceptable. The difficulty value of each test ranged from 0.61 to 0.64; the discrimination was 0.37 to 0.45; the scorers’ reliability was 0.88 to 0.93; the criterion validity based on the linkage with students’ test scores 3 months ago was 0.86 to 0.88. Overall, all of these indicators showed that these test materials can effectively measure students’ FLP.

#### Data Analysis Strategies

Correlation and regression analyses were used to evaluate the relationship between grit, FLE, CE, and FLP. All analyses were conducted using SPSS 22.0. Before statistical analyses, we analyzed the missing data in variables and found that the missing data across all items totaled less than 2.6% of possible responses. Little’s missing completely at random test was used to assess the pattern of missing data (Schafer and Graham, 2002). The results revealed that data were missing completely at random. Thus, full-information maximum likelihood estimates were employed to impute missing data for this variable.

Considering that gender and age may impact students’ FLP, these factors were controlled in the model for examining how grit, FLE, and CE affect FLP.

## Results

### Descriptive Statistics and Correlations

Table 1 shows that there were no significant correlations between age and all other variables; that gender was related significantly to grit, GLE, CE, and FLP; and that the overall correlation between grit, GLE, CE, and FLP was positive.

**TABLE 1:** Descriptive statistics and correlations for key variables.

**Variables**	**M**	**SD**	**1**	**2**	**3**	**4**	**5**	**6**
(1) Gender	1.56	0.50	1.00					
(2) Age	13.27	1.01	–0.01	1.00				
(3) Grit	27.47	6.55	0.19^∗∗∗^	–0.05	1.00			
(4) FLE	32.68	7.21	0.17^∗∗∗^	0.01	0.53^∗∗∗^	1.00		
(5) CE	87.52	23.67	0.12^*^	0.04	0.14^∗∗∗^	0.30^∗∗∗^	1.00	
(6) FLP	93.57	25.81	0.15^∗∗∗^	0.01	0.26^∗∗∗^	0.42^∗∗∗^	0.34^∗∗∗^	1.00

FLE, foreign language enjoyment; CE, classroom environment; FLP, foreign language performance. Age and gender were considered as control variables.^∗∗∗^*p* < 0.001; ^*^p < 0.05.

### Moderated Mediation Analysis

**FIGURE 1:**
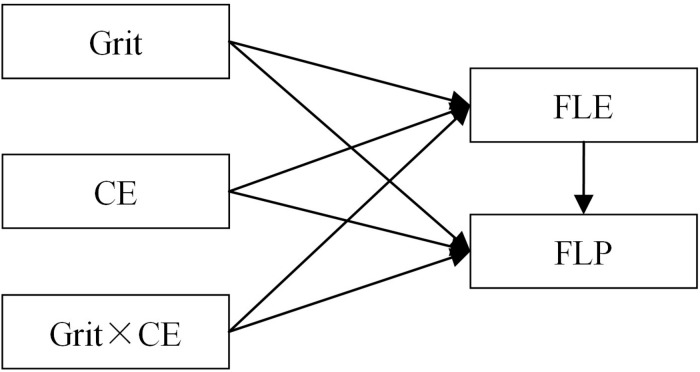
Hypothesized model. FLE, foreign language enjoyment; CE, classroom environment; FLP, foreign language performance.

We controlled for gender and age in the moderated mediation analysis. All independent variables were centered on their respective means to reduce multicollinearity between the main effects and interaction terms, and to increase the interpretability of the interaction term coefficients. Then, we followed the procedures of moderated mediation analysis of Hayes (2013) Statistical Model 15 (Figure 1), via the following steps: (a) examined the moderating effect of CE on the relationship between grit and FLP; (b) examined the moderating effect of CE on the relationship between grit and FLE; and (c) controlled the interaction item of CE and FLE, and examined the mediating role of FLE in the association between grit and PLP. Next, we conducted bias-corrected bootstrap tests with a 95% confidence interval to test the significance of the indirect effect of grit on FLP via FLE. Finally, we used the test of simple slopes to further examine the significance of the interaction effects.

The specification of these models can be seen in Table 2. Equation 1 examined the main effect of Grit and CE on FLP and the moderating effect of CE in the relationship between Grit and FLP. Equation 2 investigated the main effect of Grit and CE on FLE and the moderating effect of CE in the relationship between Grit and FLE. Equation 3 ascertained the main effect of FLE on FLP after controlling the interaction terms of CE and Grit. In each equation, students’ gender and age were included as a control variable. Grit has a significant positive predictive effect on FLP and FLE (β = 0.21/0.20, *p* < 0.001; β = 0.49, *p* < 0.001), and CE can also significantly and positively predict FLP and FLE (β = 0.32, *p* < 0.001; β = 0.20, *p* < 0.001). Thus, the interaction terms between CE and grit have significant positive predictive effects on both FLP and FLE. In addition, after controlling the interaction term between CE and grit, FLE still has a significant positive predictive effect on FLP. These results indicate that CE regulates the direct predictive path of grit to FLE and FLP, and can promote FLP through the mediation of FLE.

**TABLE 2:** Regression analysis results: Testing FLE as a mediator and CE as a moderator in the relationship between grit and FLP.

	**Equation 1**		**Equation 2**		**Equation 3**	

	**Variable (FLP)**		**Variable(FLE)**		**Variable (FLP)**	
	**β**	***t***	**β**	***t***	**β**	***t***
Gender	0.15	3.01^∗∗^	0.17	3.51^∗∗^	0.15	3.01^∗∗^
Age	0.01	0.16	0.01	0.26	0.01	0.16
Grit	0.21	4.46^∗∗∗^	0.49	11.74^∗∗∗^	0.20	4.32^∗∗∗^
CE	0.31	6.60^∗∗∗^	0.22	5.35^∗∗∗^	0.28	6.18^∗∗∗^
Grit × CE	0.34	2.36^*^	0.65	5.14^∗∗∗^	0.32	2.15^*^
FLE					0.31	5.48^∗∗∗^
*R*^2^	0.17		0.37		0.23	
*F*	17.77^∗∗∗^		48.40^∗∗∗^		20.90^∗∗∗^	

FLE, foreign language enjoyment; CE, classroom environment; FLP, foreign language performance.^∗∗∗^*p* < 0.001; ^∗∗^*p* < 0.01; ^*^p < 0.05.

In order to further test whether the mediating effect of FLE in grit on FLP is valid, the bootstrap test was carried out using 5,000 bootstrap samples from original data (*N* = 832) using the repeated random sampling method. The results showed that the 95% confidence interval of indirect effect of grit from FLE to FLP was between 0.12 and 0.26, which indicates that the indirect effect of grit through FLE to FLP is valid.

**FIGURE 2:**
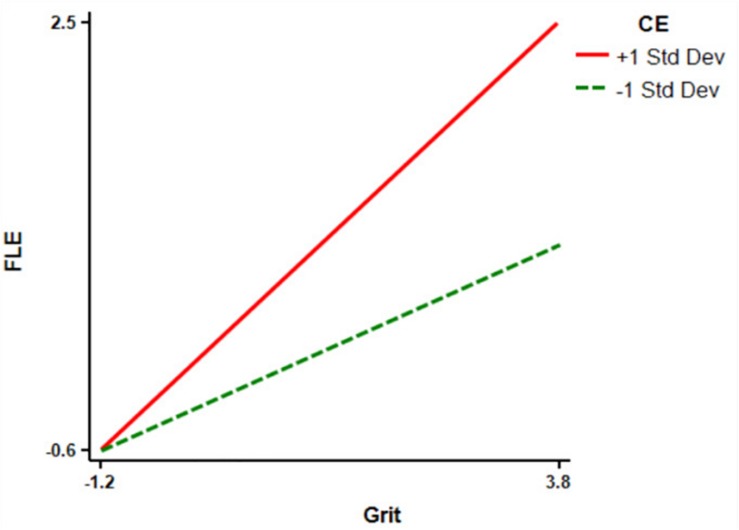
Relationship between grit and FLE at different levels of CE. FLE, foreign language enjoyment; CE, classroom environment.

**FIGURE 3:**
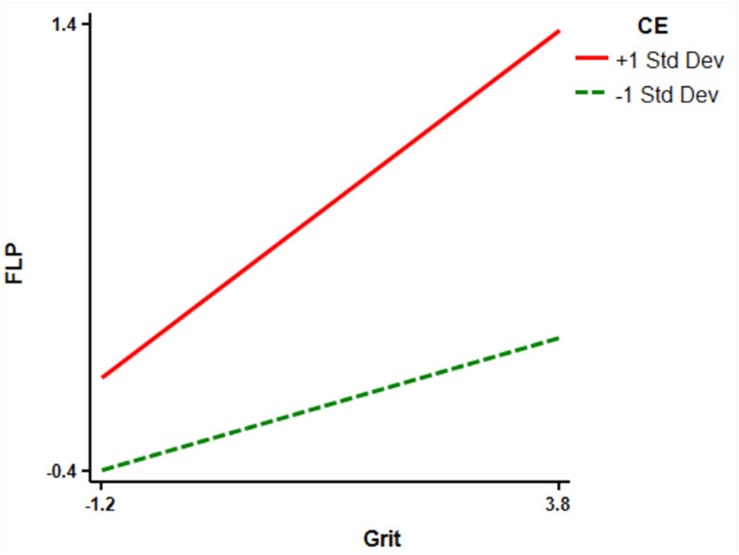
Relationship between grit and FLP at different levels of CE. CE, classroom environment; FLP, foreign language performance.

Finally, in order to clarify the significance of the moderating effect of CE in the relationship between grit, FLE, and FLP, CE was divided into high and low groups by adding and subtracting a standard deviation from the average. Then, the simple slope test syntax of Schubert and Jacoby (2004) was adopted for the simple slope test. The results showed that the grit of the high CE level group had a positive predictive effect on FLE (simple slope = 0.62, *t* = 13.34, *P* < 0.001), which was higher than that of the low CE level group (simple slope = 0.29, *t* = 5.43, *P* > 0.05) (Figure 2). In addition, the positive predictive effect of grit on FLP in the high CE level group (simple slope = 0.28, *t* = 5.31, *P* < 0.001) was higher than that in the low CE level group (simple slope = 0.11, *t* = 1.72, *P* < 0.01) (Figure 3). This result shows that the positive predictive effect of grit on FLE and FLP increases with the increase of CE level. According to these results, then, it can be concluded that there is a mediating regulatory effect in the relationship between grit and FLP.

## Discussion

This study examined the effect of grit on FLP through FLE and CE from the perspective of PosPsy. After controlling for gender and age, this study found that grit had a direct positive effect on FLE and FLP, which was consistent with previous studies (Perkins-Gough, 2013; Macnamara et al., 2014). Grit is an important positive psychological trait. Indeed, individuals with a high level of grit are thought to be able to maximize their abilities because they have higher concentration and are less discouraged by failures and setbacks (Credé et al., 2017). In the process of foreign language learning, individuals struggle to avoid various setbacks. This is especially true for middle school students, whose foreign language ability is still in the lower levels. Given that, many students’ foreign language academic self-efficacy is low (Huang, 2012), while their foreign language anxiety level is high (Pappamihiel, 2002). In turn, this can make students put less effort into foreign language learning, therefore making it difficult for them to succeed in a foreign language at school. However, students with a high level of grit will persist in overcoming difficulties in foreign language learning and eventually achieve good academic performance (Aparicio et al., 2017; Lan and Moscardino, 2019).

This study also found that grit had a positive effect on FLP through FLE, consistent with our hypothesis. Previous studies had found that Chinese students have lower positive emotions in foreign language learning and higher levels of foreign language anxiety than American students (Dewaele and MacIntyre, 2014). One possible reason may be that most middle school students in China lack interest in learning foreign languages. Rather than being intrinsically motivated, most Chinese students learn foreign language mainly to pass exams and enter higher education (You and Dörnyei, 2016). Chinese students also face great pressure from their parents and social environment. In Chinese culture, education and filial piety are strongly emphasized (Essau et al., 2008). Children should try their best to meet their parents’ requirements – especially with respect to a high academic performance. Therefore, for Chinese middle school students, grit plays an important role in their FLP by improving their FLE. Middle school students with high grit are able to overcome difficulties and devote themselves to foreign language learning even under great external pressure.

According to the social cognitive theory of self-regulation (Bandura, 1991), students will evaluate their own behaviors, thus triggering positive or negative emotional reactions. When they make enough effort in foreign language learning, they tend to positively evaluate their own behaviors, thus producing positive emotional reactions, such as FLE. Enjoyment is a positive emotional feeling that stems from breaking through homeostatic limits and stretching beyond oneself to accomplish something difficult (Dewaele and MacIntyre, 2016). Both the broaden-and-build theory and the control-value theory emphasize the positive predictive effect of positive emotions on academic performance (Fredrickson, 2001; Pekrun et al., 2007). FLE can expand individual cognitive resources and make students’ learning more efficient. In addition, FLE can also help learners gain positive power, relieve the pressure on foreign language learners, and promote their interest in foreign language learning (Piniel and Albert, 2018).

This study also found that CE has a significant positive predictive effect on FLP, consistent with previous studies (Baek and Choi, 2002; Patrick et al., 2007). In a positive CE, students can participate in classroom activities more actively, and teachers pay more attention to students (Anderson et al., 2004). Conversely, in a negative CE, teachers spend more time maintaining classroom order, and students are more easily distracted by the external environment. Dewaele and MacIntyre (2014) found that positive classroom activities could boost FL learners’ levels of FLE.

Another interesting finding of the present study is that CE moderated the relationship between grit and FLE, and between grit and FLP. The results of the simple slope test showed that, in a good CE, students’ FLE and FLP increase significantly with the increase of grit. However, in a poor CE, students’ FLE and FLP did not increase significantly with grit. This result indicates that, although grit is a positive personality trait that can improve academic performance, in a negative CE, the influence of grit on academic performance will be reduced. Previous studies had found that grit does not always play a significant positive role in the prediction of academic performance (Macnamara et al., 2014; Paunesku et al., 2015; Steinmayr et al., 2018). One possible reason is the influence of environmental factors. In line with that, this study clarified that a good CE is an important factor in how positive personality traits, like grit, relate to academic performance. Additionally, in the present study, in a negative CE, the impact of grit on FLE was not significant. Previous studies have found that students’ academic emotion is largely influenced by the CE; for instance, in a negative CE, even students with a high level of grit cannot enjoy their studies (De Smet et al., 2018).

In addition, gender and age as control variables also impacted the results. This study found that there was no significant correlation between age and our study variables. One possible explanation is ascribed to the homogeneity of participants. Although the age range of participants in this study was relatively wide, most students were between 12 and 14 years old in middle school. Moreover, this study found that gender was significantly positively correlated with Grit, FLE, CE, and FLP, namely females reported higher scores in those scales. Regression analysis also showed that gender had significant predictive effect on both FLP and FLE. The results indicated that we should pay more attention to the FLP of males, which can promote FLP by enhancing their Grit, FLE and CE.

The present study has some limitations. First, the study controlled for only two variables, gender and age. Other factors may also affect students’ academic performance. Second, all measures except FLP were based on adolescents’ self-report. The self-report of grit is not always reliable especially with such a young sample. Future research should consider gathering data through multiple methods. Third, during middle school, it is a transitional period from pre-adolescence to adolescence. As physical and psychological features dramatically develop, there is a possibility that FLP impacts the mechanism, which is contingent on developmental stage of adolescence. Future research can further differentiate the similarities and differences of FLP influencing mechanism between pre-adolescents and adolescents.

Despite the limitations, the findings still have important implications for foreign language learning for middle school students in China. The study results indicate that grit not only directly promotes the FLP of middle school students but also indirectly improves FLP by promoting FLE. Additionally, in the context of positive CE, the impact of grit on FLE and FLP increases, but in the context of negative CE, grit does not have any effect on academic performance at all. Teachers should pay attention to the cultivation of students’ positive personality traits (such as grit) in order to stimulate students’ positive academic emotions (such as FLE) and, at the same time, create a good learning environment (such as CE). In this way, students’ academic performance can be improved.

## Data Availability

The datasets generated for this study are available on request to the corresponding author.

## Ethics Statement

This study was carried out in accordance with the recommendations of The Ethics Committee of Shihezi University with written informed consent from all subjects. All subjects gave written informed consent in accordance with the Declaration of Helsinki. The protocol was approved by The Ethics Committee of Shihezi University.

## Author Contributions

HW conceived the study, drafted the manuscript and revised the manuscript critically for important intellectual content. KG participated in and supervised data acquisition, drafted, and modified the manuscript. WW developed the study design, participated in and supervised data collection, performed the statistical analysis, and drafted the manuscript. All authors gave their final approval of the current version of the manuscript.

## Conflict of Interest Statement

The authors declare that the research was conducted in the absence of any commercial or financial relationships that could be construed as a potential conflict of interest.
